# Notes and News

**Published:** 1893-06-17

**Authors:** 


### Notes and News.

COLLECTED AND COMMUNICATED.

THE London Homœopathic Hospital is rapidly disappearing, and the rebuilding on a much extended site will be commenced with all possible dispatch. The Duchess of Teck and Princess May have promised to lay the foundation stone in July, but the Royal Betrothal may necessitate some modification of this arrangement. The executive have already obtained £30,000 towards the cost of the new buildings, but another £10,000 probably will be required to complete. Two houses in Powis Place—adjoining the old building—have been fitted up as a temporary hospital, and will accommodate 40 in-patients. The out-patient department also will be carried on without any interruption. The accepted designs for the new hospital are at present exhibiting at the Royal Academy. It will provide about 100 beds.

THE Royal Victoria Hospital at Montreal is an imposing addition to the medical charities of that city. The part now erected comprises the medical, surgical, administrative, and nursing departments. The hospital has been erected through the munificence of Lord Mount Stephen and Sir Donald Smith, and will be a monument worthy of the illustrous founders. The buildings are situated on the steep eastern slopes of Mount Royal, overlooking the city and the St. Lawrence River; mountains form the back ground. Fine as is this situation the group of buildings is limited to a plot of 325 ft. The English architect chosen to erect the buildings has had some difficulties to contend with. The main buildings will, when completed, comprise thirteen distinct blocks connected by bridges. The arrangement of the buildings is irregular. They form three parallel groups of irregular lengths, buildings running straight across and uniting them. There are good consulting rooms, operation rooms, and accommodation for the staff. Special arrangements are made for paying patients. The hospital for infectious diseases has been kept quite distinct. We propose to give a more detailed account of the institution at a later date.

THE twenty-seventh annual meeting of Dr. Barnardo's Homes was held in the Albert Hall on the 7th inst., Lord Brassey in the chair. The immense building was well filled, and the programme was varied and interesting. It was, in fact, a perfect example of what can be accomplished by discipline and organisation, but it could be desired that these meetings did not require the attendance of the little people at so late an hour. We regretted to notice a crowd of these tired little folk standing waiting for a train for about half-an-hour. When we left at eleven o'clock they were still there. Some of the facts given in the Chairman's address were almost startling. The income for the past year (1892) was £133,000; the Homes comprised 51 different institutions, containing nearly 5,000 children; the baker's bill alone amounts to £150 per day; 2,730 fresh cases were admitted during the year, the average for every day of the year being nine. The hospital in connection with the Homes (Her Majesty's Hospital) contains 74 cots, 59 of which have been occupied. The in-patients numbered 758; out-patients, 4,328. Each cot costs on an average £30 per annum, and of these 16 have been supported by special gifts from friends, and 15 by the "Young Helpers' League."

A CONTEST is being carried on between the Committee of the Radcliffe Infirmary, at Oxford, and the City Coroner, a Mr. Hussey. It appears that on six occasions within the last six months the Coroner has ordered the removal of the bodies of patients, who have died in the Infirmary, to the City Mortuary, which is at some distance from the Infirmary. These removals have not been made for the purpose of holding *post mortem* examinations, but for the purpose of the Coroner's inquest. The Committee maintain that these removals are illegal, on the ground that the Coroner has no power of removal except for the purpose of burial or *post mortem* examination; and they argue that he is bound to hold his inquest and that the jury should view the body at the Infirmary, where the death took place, and where the witnesses are present. Having taken the opinion of counsel, they were advised that their view of the law was correct, and that they should lay the facts before the Lord Chancellor. A petition was accordingly presented to the Lord Chancellor, praying him to intervene if he was of opinion that the action of the Coroner was illegal. The Coroner, at the same time, laid his view of the matter before the Lord Chancellor, maintaining that he is within his rights in removing the bodies. He also stated that he was not treated with courtesy by the medical staff at the Infirmary, and that, moreover, "the bodies in the keeping of the officers of the Infirmary *are not safe from violence*," meaning, it is presumed, that unauthorised *post mortem* examinations have been held upon them. The Chancellor's answer to the petition is now published. His Lordship states that while he does not think it desirable to express an extra judicial opinion on the subject, he has informed the Coroner that "in assuming such a power the Coroner should be guided by the consideration whether grave public inconvenience would follow from any other course." This, we presume, is a hint that the inquests should in future be held at the Infirmary. Here the matter rests in the meantime; but it is expected that the question will be brought into a court of law, when the decision will be awaited with some interest by the medical profession.

